# 

**Published:** 1893-10

**Authors:** 


### CONTENTS.

ORIGINAL ARTICLES—

Abstract of Paper Read by Reginald H. Sayre, M, D., before the Pan American Medical Congress, Washington, Sept. 1893...... 515

Note on the Operation of Circumcision. By N. Davies-Colley... 518

Report of case of Twin Pregnancy with Distinct Placenta, one of which was a Placenta Prævia. By Fred V. Turk, M. D., Stilesboro, Ga.......... ........................................ 552

Antiseptic Vaginal and Intra-Uterine Injections unnecessary, if not injurious in the daily practice of Obstetrics. By Darwin Colvin, M. D., Clyde, N. Y........................................ 523

SOCIETY REPORTS—

Transactions of the Atlanta Obstetrical Society................ 529

(Continued on Next Page.)

EDITORIALS— ........................................ 559-562

An Open Letter. Tri-State Medical Society of Alabama, Georgia and Tennessee.

CORRESPONDENCE—

A Plagairism Exposed. By J. M. Thomas, M. D., Atlanta, Ga.. 538

BOOK REVIEWS— ........................................ 549

SELECTIONS AND ABSTRACTS—

The Treatment of the Gouty State. Clinical Lecture Delivered by Frank Woodbury, M. D. ........................................ 551

The Treatment of Intestinal Hemorrhage in Typhoid Fever..... 555

Treatment of Loss of Sexual Power by Ligation of Veins........ 556

Diarrhea of Children........................................ 558

PRESCRIPTION DEPARTMENT—........................... 564–565

SPECIAL NOTES—........................................566–572

